# Training Primary Health Professionals in Breast Cancer Prevention: Evidence and Experience from Mexico

**DOI:** 10.1007/s13187-016-1065-7

**Published:** 2016-06-30

**Authors:** Laura Magaña-Valladares, María Cecilia González-Robledo, Cynthia Rosas-Magallanes, Miguel Ángel Mejía-Arias, Héctor Arreola-Ornelas, Felicia M. Knaul

**Affiliations:** 1 Secretaría Académica, Instituto Nacional de Salud Pública, México, Universidad 655, Col. Santa María Ahuacatitlán, C.P. 62100 Cuernavaca, Morelos Mexico; 2 Centro de Investigación en Sistemas de Salud, Instituto Nacional de Salud Pública, México, Avenida Universidad 655, Col. Santa María Ahuacatitlán, C.P. 62100 Cuernavaca, Morelos Mexico; 3 Competitividad y Universalidad en Salud, Fundación Mexicana para la Salud, A.C., Periférico Sur 4809, El Arenal Tepepan, Tlalpan, 14610 México, DF Mexico; 4 Tómatelo a Pecho, A.C. Periférico Sur 4809, El Arenal Tepepan, Tlalpan, 14610 México, DF Mexico; 5 Miller School of Medicine, University of Miami, 1601 NW 12th Ave, Miami, FL 33136 USA

**Keywords:** Face-to-face learning, Blended learning, MOOC, Breast cancer, Mexico, Training courses, Virtual education and multidisciplinary training, Health promoters

## Abstract

To analyze the key successful factors of a national educational strategy for early breast cancer detection developed in Mexico for primary health care personnel from 2008 to 2014, an educational strategy to train physicians, nurses, health promoters, and medical students from local ministries of health with a competency-based approach was developed and implemented using diverse educational modalities, face-to-face, blended, and a massive open online course (MOOC). Formative and summative evaluations were used during the implementation of the course. A total of 19,563 health professionals were trained from 2008 to 2014. The graduation rate, an average of all educational modalities, was 91 %, much higher than those previously reported in the literature. The factors that might have influenced this success were (1) the training strategy, which was designed according to the characteristics and specific needs of the target groups; (2) the political will and commitment of the country’s health authorities; (3) the technological and educational models used; and (4) the punctual follow-up of participants. This study shows that carefully designed educational interventions can improve service professionals’ competencies and that regardless of the modality, face-to-face, blended learning, or MOOC, high graduation rates can be achieved. Further evaluation is required to demonstrate that the competencies remained in all target groups after 6 months of the intervention and that the women served by the trained personnel were provided accurate information and timely diagnoses of breast cancer.

**Electronic supplementary material:**

The online version of this article (doi:10.1007/s13187-016-1065-7) contains supplementary material, which is available to authorized users.

## Background

Breast cancer (BC) is a leading cause of death and disability among women around the world, affecting both high- and lower-income countries. In total, 53 % of BC cases and 64.2 % of deaths occur in low- and middle-income countries (LMICs) [1]. In Mexico, BC is the second leading cause of death among women aged 30 to 54 years and affects all socioeconomic groups. BC mortality-adjusted rates increased from 6.2 per 10,000 women in 1980 to 9.0 per 100,000 women in 2013 [2]. Early detection continues to be one of the cornerstones of BC control efforts [3], yet in Mexico, the majority of cases are detected late. Only an estimated 10 % are detected in stage I, when the probability of a cure with adequate treatment is likely.

Late detection is related to a lack of knowledge of the benefits of detection and treatment and the scarcity of adequate facilities for screening and diagnosis [4]. Other factors such as insufficient and inadequate information about the disease provided by primary health care personnel and embarrassment associated with the clinical examination may result in a delay, in the lack of participation in the screening processes, or in the search for health care despite the presence of symptoms [5, 6].

In Mexico, the lack of properly trained primary health care personnel constitutes a barrier to accessing breast health education and screening and hence to timely treatment. Villarreal et al. published a study on the training of medical students and residents in cancer screening and demonstrated a lack of basic knowledge. The authors of the study concluded that inadequate medical counseling for cancer detection can generate a lack of awareness in the patient of the need for early cancer detection [7]. González-Robledo et al. [8] conducted an assessment of the non-specialist, basic medical school curriculum, which is the level of initial training for primary-care professionals, to identify how physicians and nurses are trained in BC care in the course of their undergraduate studies. Most of the curricula lacked BC training components, and the authors recommended strengthening undergraduate physician and nurse training to include health promotion regarding BC risk and early detection [9]. Keating et al. [10] presented the results of the training program described in this paper in its application with health promoters in several Mexican states. The evaluation of knowledge uptake among both professional and community health promoters showed statistically significant increases in knowledge of BC risks and early detection methods.

A priority in addressing the challenge of BC in LMICs such as Mexico is to ensure a properly trained primary health workforce as well as to provide breast health education for women in their communities. In response to this need, the National Institute of Public Health (NIPH), the civil society organization Tómatelo a Pecho, A.C., the Harvard Global Equity Initiative, and the Harvard Medical School through the Global Task Force for Expanded Access to Cancer Care and Control (GTF.CCC) program, with implementation funding from the National Commission for Social Health Protection of Mexico, developed an extensive and rigorous training program for primary health care personnel (physicians, nurses, medical students in social service, and health promoters).

The courses included basic information regarding the epidemiology of the disease, training on breast health knowledge for women and on breast clinical examination (BCE), and an introduction to the treatment and management of the disease.

Training methodologies were developed to enable effective access to the target populations. The incorporation of Communication and Information Technologies (CITs) [11] and the creation of virtual learning environments together with a competency-based education [12] were considered to be suitable strategies to reach a significant number of health care workers; many of whom were employed in primary clinics and could not leave their posts for any significant lengths of time. These methods offered flexibility in the timing of the training as well as the location [13].

Online education was reinforced by short in-person trainings that were focused on BCEs. A series of studies spanning several countries reported that these types of training programs improved knowledge of BC management among health care personnel.

Trovato et al. [14] taught a virtual course for nurses and found that innovative methods were necessary to achieve an effective educational approach to teaching breast cancer-related content. Arving et al., based on a training of nurses, found that students highly valued blended learning because it could be accessed at any time. Nurses also noted the importance of the interaction between the students and teachers [15].

This article describes the strategies and actions developed within a training program for early breast cancer detection that was designed for primary health care personnel—health promoters, nurses, general physicians, and medical students—and implemented in the following states of Mexico: Morelos, Jalisco, Nuevo León, and Puebla, and at a national level, for the Mexican Social Security Institute (IMSS) between the years 2008 and 2014. It also describes the achievements and challenges in the design and implementation.

## Methodology

The training strategy was designed, developed, and implemented by an interdisciplinary and multidisciplinary group including (1) experts on breast cancer (professors/researchers, physicians, and oncologists), (2) a team of instructional designers with a pedagogical profile with experience in distance education and expertise in educational technology, (3) a team of graphic designers with experience in images and communication for health, (4) a team of web programmers with expertise in technological learning platforms and HTML language, and (5) a multimedia producer and specialists in multimedia communication and programming and in video and audio editing.

The design of the training strategy included (1) the identification of target groups (who needs to know about early detection strategies), (2) the identification of the competencies that these target groups should have to be successful (knowledge, skills, and attitudes), (3) the analysis of the specific conditions and needs of the participants (learning styles, time availability, disposition), and (4) the perception of the political will to support a national training strategy.

The identified target groups were composed of the following primary health professionals: physicians, nurses, health promoters, and medical students who were part of the primary health system. The identified general competencies that all of them should have are shown in Tables 1 and 2. The specific competencies for each target group are described in Annex 1.

**Table 1:** General competencies and courses’ content

Competency	Course content
1. Develop health promotion actions to reduce the risk of health loss	• Know your body, anatomy and physiology of the mammary gland
	• Risk factors for the development of breast cancer
	• Warning signs for breast disease
2. Implement evidence-based actions of breast cancer early detection	• Self-examination
	• Clinical examination
	• Mammography screening
3. Identify breast abnormalities	• Benign diseases
	• Carcinogenesis in breasts
4. Identify referral processes to be used in each case to provide adequate and timely guidance to those affected by the disease	• Referral process
	• Health system institutions and services
	• Communication and patient follow-up
5. Identify the institutions and organizations that provide multidisciplinary support to the relatives of women with breast cancer	• Services available to support the relatives
	• Breast cancer in the family
	• Return to everyday life

**Table 2:** Between 2010 and 2014, 19,563 health professionals were trained with 66 courses using 3 modalities

Participants	Face-to-face		Blended		MOOC		Total
	Register	Graduate	Register	Graduate	Register	Graduate	
Physicians and nurses	4,542	4,542	1,417	1,049	11,569	10,191	17,528
Medical students	1,617	1,617	–	–	–	–	1,617
Health promoters	–	–	418	309	–	–	418
Total number of participants per modality	6,159	6,159	1,835	1,358	11,569	10,191	19,563
Total graduation rate		100 %		74 %		88 %	91 %

Different educational modalities were considered for each target group according to their profiles, learning styles, characteristics, training needs (availability, time, and disposition), and the general and specific competencies. During the planning process and according to the modality identified for each target group, face-to-face, blended, or massive open online course (MOOC), a vast range of multimedia materials were developed with a thorough follow-up plan to ensure participant course completion.

To measure the development of competencies, a “cube of competencies” was designed, in which activities, topics, and knowledge levels were related (according to Bloom’s taxonomy). This design allowed for the development of the competencies to be ensured as the student followed and developed the course (Annex 2). The description of the educational intervention of each target group was as follows:

1. General physicians and nurses. For this target group, a 40-h blended course was developed. An initial 8-h face-to-face course was conducted that focused on the theoretical and practical training of clinical examinations of breasts and clinical diagnoses. This course was followed by a 32-h online course that covered the following topics: epidemiology and timely detection of breast cancer, clinical profile references, counter-reference mechanisms, integral management, and follow-up of the patient. A manual for this course was developed and used, as further described.
2. Health promoters. For this target group, a 40-h course was prepared with a face-to-face workshop of 8 h followed by an online course of 32 h. The topics addressed in the face-to-face workshop were the theoretical content related to breast self-examinations and clinical examinations of the breasts, practical breast self-examination, and guidelines to working with communities.
  The content of the virtual course included breast cancer, a priority for the health of Mexican women and of Mexico, how to carry out timely breast cancer detection, characteristics of the breast (normality and abnormality), breast cancer diagnosis and treatment, the return to everyday life, and effective pedagogical communication with communities. A unique feature of this target group was that a practicum experience was integrated into the course, in which the participants were required to organize and conduct a 10-h workshop in the community in order to be accredited with the course. An educational kit, later described, was developed for this purpose.
3. Medical students. A 6-h face-to-face course was prepared for this target group, including the following topics: breast cancer clinical and self-examination techniques, strategies to prevent breast cancer, and reference and counter- reference mechanisms.

The educational strategy was designed using a competency-based approach with an emphasis on (1) student-centered activities, (2) learning environments based on the constructivism theory [16], (3) innovation, and (4) performance of the teacher as a facilitator of learning by applying technological and educational methodologies that favored collaborative work and problem solving [17, 18].

The training program was oriented towards the active involvement of the participants in the construction of their own learning through activities, in which they applied the acquired knowledge in practice (Annex 3). Collaboration and self-directed learning using electronic media was emphasized. The use of this approach was fundamental for the development of competencies, which was based on learning, learning to do, and learning to be. In this way, personal experiences were reconsidered and the new information obtained by the participants originated from their own perspective and within a determined context; accordingly, practice was fundamental for significant learning [19].

The training strategy was first implemented in the following four Mexican states: Morelos, Jalisco, Nuevo León, and Puebla. These were intentionally selected based on the following criteria: (a) geographical diversity; (b) rates of prevalence, incidence, and mortality of different types of BC; (c) previous evidence of the development of governmental action regarding early BC detection; and (d) interest of the local agents in participating in the training strategy.

The selection of participants who received the training was made by state health authorities according to the following criteria: belonged to the primary health workforce (physicians, nurses, health promoters) and had internet access and sufficient availability of time (approximately 10 h a week for 1 month).

In the second stage, the challenge was to reach, at a national level, all the physicians and nurses of the Mexican Social Security Institute, which were more than 10,000 and were also geographically disperse. The training strategy for this stage included the development of a MOOC to reach all physicians and nurses who composed the primary health personnel of the Mexican Social Security Institute (IMSS in Spanish) at the national level.

The course design was automated and self-directed, seeking complete interaction with the content by using multimedia resources to promote learning styles. The MOOC was accompanied by animated videos, interactive exercises, and assessments of learning for each module.

It was decided to implement game theory (gamification) for different activities as an attraction and motivating tool for the participants; this consisted of a “reward” system for each successfully concluded activity, a competition between participants through an online ranking that allowed participants to see and compare their progress, and socialization of their achievements.

The top results from the participants, at the delegation level, were published on a competition web board for the strategy. The MOOC’s implementation was accompanied by a 3-h face-to-face workshop that reinforced some topics and motivated participation. Similarly, a very cautious participant follow-up strategy was designed to maintain contact with the participants using motivational messages to ensure that they concluded the course. IMSS authorities were very involved, demanding the participation of all professionals. Weekly compliance reports were made, which exhibited the progress of each state on the platform.

The following materials were specifically designed and developed for the two stages of the training strategy:

1. A manual for physicians and nurses. The manual was designed for health professionals whose work was crucial in the early detection of signs that suggest breast cancer. The objective of this manual was to provide tools to identify and follow-up a suspected case of breast cancer, according to the health care system in which the participants worked. All physicians and nurses who participated in the educational strategy received a printed copy.
2. An educational kit for health promoters that included
  - A manual for health promoters that was developed and designed for personnel involved in community health care. The main objective of this material was to provide information and tools to improve early breast cancer detection. The manual allowed participants to generate ideas on how to communicate each topic with simple and clear language.
  - A flip chart for the workshop sessions. These were visual support aids for health workers in the community to use during the presentation of community issues. These data allowed participants to understand the magnitude of the problem, the aspects of self-care, and the importance of breaking myths and stigma and knowing one’s own body, which can contribute to the early and timely detection of breast cancer.
  - A guide for the development of practical workshops in the community. This included educational aspects to be considered when developing workshops in the community.
  - A marathon game. This was a recreational resource that incorporated the fundamentals of a game of marathon, in which participants used dice and questions to progress towards a goal. The game contained questions related to the identification of warning signs, breast self-examination techniques, treatment, and return to daily life.
  - A memory match game, which was a recreational resource that allowed users who were involved in medical consultation and breast self-examination techniques to identify warning signs that could suggest breast cancer. The goal, achieved through cognitive effort, was for participants to match pictures of injuries and self-examination techniques to each of their corresponding names.
  - Self-exploration recording sheets. These provided a visual guide that helped women record their monthly findings.

The online component of the course provided the following resources: (a) audio-visual materials, multimedia presentations, animated resources, and videos and (b) scientific readings.

The Moodle® platform was used as the learning management system (LMS). This platform integrates the learning environment through course content, topics of communication (forums, chats), and follow-up (student monitoring and evaluation) tools.

The effectiveness of the training strategy was evaluated. To measure the course’s effectiveness, a minimum accreditation of 80 % of the competencies was established. This was measured by the formative and summative evaluations included in the course. In addition, a PRE and POST test external evaluation was implemented to the health promoters group to measure the knowledge change through the training strategy; the first results were presented by Keating et al. 2013 [10].

## Results

The first result of the training strategy was the identification of the competencies that the physicians, nurses, health promoters, and medical students were required to have to provide accurate information to women and timely diagnoses of breast cancer (Annex 1). Based on the identified competencies, a training strategy was developed, producing diverse modules and resources as further described.

The graduation rate of the participants who completed and passed the final evaluation was 91 %, with the following distribution by profession: 90 % physicians, 86 % nurses, 69 % health promoters, and 100 % medical students.

The course for health promoters was designed with an in-field component, in which they had to replicate the course in their communities. A total of 3500 women from various communities were estimated to have received the training, as the health promoters were instructed to replicate the training in a 10-h workshop under the supervision of field coordinators. In an external evaluation of the health promoters’ course, post-training surveys demonstrated an increase in treating BC as a problem, with participants understanding the screening, treatment, and coverage issues and gaining knowledge of breast cancer risk factors, symptoms, and components of a family history. These competencies were maintained up to 3 and 6 months after the training [10].

## Discussion

Key factors can be identified that were influential in accomplishing an effective training strategy as measured by the high graduation rates, an average of 91 % including all modalities and 81 % excluding face-to-face:

1. A solid educational and technological design that was grounded in the development of competencies and in adult learning theory and took into consideration the specific needs and characteristics of the target groups. This design emphasized
  - Situated learning because it was closely linked to the context, such as the intervention activity with the health promoters group that facilitated learning by encouraging the participation and commitment of participants through the proposed educational activities.
  - Designing relevant and immediately useful materials (described in the “Results” section) that led to the development of new knowledge.
  - Developing an enabling environment for participation and collaborative learning, which was implemented in each of the different methods (face-to-face, b-learning, and online) according to the participants’ characteristics.
  - Adopting a holistic approach in the design with activities that developed and improved knowledge (theoretical/participatory activities), skills (breast self-examination practice and clinical breast examination), and attitudes (empathy towards patients’ family and communities).
2. Punctual follow-up and monitoring of participants. This point was very relevant to achieving the results. Weekly monitoring of participation, motivational messages, and a competitive strategy that compared the involvement of the different states in Mexico were used to monitor the students’ progress.
3. Political will and commitment of Mexican health authorities and public policy makers who understood the urgency of taking effective actions to control breast cancer.

The identification of problems and needs, regarding the personnel assigned to services, had been reported in studies such as those of [7, 20, 21], which concluded that (1) trained human recourses for treating cancer were insufficient for the country, (2) the knowledge that physicians and nurses had in cancer detection was suboptimal, and (3) there were delays in health care due to factors inherent to women (fear and ignorance of the disease) and to health service factors (insufficient training of general physicians and gynecologists in the detection of breast cancer).

In this sense, health authorities have shown a clear interest in developing actions since the beginning of the millennium, both in policy development and infrastructure, to improve the care and training of health personnel to enable better responses to the population’s particular needs. Regarding this last point, government stakeholders in partnership with civil society organizations and academia have developed training initiatives for primary health care staff to improve the early detection of the disease.

It became evident that the competency-based approach, with the practical component of using games to engage participants, was very effective in training people in the community and officials with community responsibilities. Moreover, MOOCs are undoubtedly an effective training strategy as long as they are monitored closely and have the following and commitment of authorities in supporting health staff and motivating them to conclude the course, as evidenced by this intervention.

The evidence from this experience shows that, to increase the competencies of health personnel, training strategies must follow a holistic approach, including not only physicians and nurses but health promoters, medical students, and the community.

The biggest challenges regarding this strategy were found in the health promoter course due to the extremely varied profile of the participants. Their educational level was uneven, ranging from people with academic degrees to midwives who had never used a computer. Nevertheless, the results were surprising in that despite these difficulties, the participants successfully finished the course, including the last activity, which was the preparation of the 10-h workshop for the community.

In this study, it was shown that an integrated approach that considered all public health professionals and actors was the best approach to coordinate action. However, to make learning significant, the courses had to be specific to the target group and include diverse learning-teaching strategies to engage the students.

It was specifically proven that blended learning, with a practical component of using games to engage the participants, was effective in training health promoters and in reaching communities. Physicians and nurses achieved high levels of success in an e-learning environment with very little face-to-face intervention.

A lack of basic knowledge and skills, due to a large gap in their curriculum in relation to this topic, was observed in the medical interns. There is an urgent need to include thorough and in-depth BC detection in all health-related professional programs.

The MOOCs showed the highest completion rate reported in the literature [22], and this achievement was due to the key factors explained earlier, in particular the monitoring of students and the strong support from the authorities, which were particularly important to this modality.

Finally, the lessons learned in this training strategy can be replicated in other similar settings to generate more evidence of its results. Additionally, further research is needed to evaluate whether the achieved competencies of the health professionals made an impact on early breast cancer detection in Mexico.

## Electronic Supplementary Material

ESM 1 (DOCX 13 kb)

ESM 2 (DOCX 65 kb)

ESM 3 (PDF 2580 kb)
