# Supplementary material for: Training Primary Health Professionals in Breast Cancer Prevention: Evidence and Experience from Mexico
Source: J Cancer Educ. 2016 Jun 30;33(1):160–6. doi: 10.1007/s13187-016-1065-7 (PMC5762772; doi:10.1007/s13187-016-1065-7)
Supplement: Supplementary file 1 — (DOCX 13 kb) [file 13187_2016_1065_MOESM1_ESM.docx]

**Specific courses' competencies**

**1.- Health promoters:**

After completing the course, the participant will be able to:

1. Develop group communication and management strategies to communicate the course's content in communities.
2. Identify the reasons why breast cancer is a health priority in Mexico and the rights provided by the law to ensure free care services for women with breast cancer.
3. Perform breast self-examination techniques and breast clinical examinations to recognize warning signs and motivate women to seek timely help in the presence of any signs of abnormality.
4. Apply breast cancer promotion and early detection practices in daily practice through self-examination and clinical examination to reduce the risk of health loss.
5. Explain the main myths and beliefs related to breast cancer to reduce the stigmatization of women who suffer from breast cancer.
6. Identify and tackle the different barriers and obstacles that delay the early detection and early diagnosis of breast cancer in their community; provide support and advice to women.
7. Actively participate in communication and information activities, along with local health institutions, to promote the effective diffusion of breast cancer early detection practices.

**2.- Physicians and nurses:**

After completing the course, the participant will be able to:

1. Identify the activities that make their practice a fundamental component of the detection, diagnosis and treatment of breast cancer.
2. Incorporate breast cancer promotion and early detection actions in daily practice to reduce the risk of health loss.
3. Perform the proper techniques of clinical examinations of the breast glands for early breast cancer detection.
4. Identify referral and counter-referral mechanisms to be used in each case to provide adequate and timely guidance to women affected by this disease.
5. To recommend actions, from a medical point of view, that promote healthy lifestyles.
6. Recognize the institutions and organizations aimed at providing multidisciplinary support to families with breast cancer patients.
