# Supplementary material for: Training Primary Health Professionals in Breast Cancer Prevention: Evidence and Experience from Mexico
Source: J Cancer Educ. 2016 Jun 30;33(1):160–6. doi: 10.1007/s13187-016-1065-7 (PMC5762772; doi:10.1007/s13187-016-1065-7)
Supplement: Supplementary file 2 — (DOCX 65 kb) [file 13187_2016_1065_MOESM2_ESM.docx]

**Competencies cube**

The competencies cube was created to establish consistency in the instructional design of the online courses. The cube integrated the learning outcomes, content, and cognitive level acquired with the competencies by identifying the need and type of evidence for each of the intersections.

Three elements

- Learning outcomes (competencies)
- Course's topics or content blocks
- Cognitive levels

Learning outcomes indicate the specific competencies obtained per module, as general competencies correspond to the entire course. Topics or content blocks are organized by the teachers according to how they should be addressed. Mapping should only be performed with the first level of the syllabus, although some subtopics are worth including for greater certainty of what will be covered in each block. Cognitive levels correspond to Bloom's taxonomy and are grouped into the following five levels:

1. **Knowledge/understanding:** Although Bloom's taxonomy handles these two levels separately, it must be ensured that the participants not only learn or access new information but that they retain it, considering the fact that they had previous information to encode new knowledge and that they must reorganize it based on their personal interpretations to establish casual relationships.
2. **Application**: This cognitive level should consider all of the activities that enable the use of new knowledge (personally redefined) in a given situation. Problem solving techniques, cases or scenarios can be considered, as well as instruments or tools to address different situations.
3. **Analysis:** Activities at this level should be oriented to the identification process or element classification, i.e., the relationship between them and the organizing principles that govern the coexistence of these elements.
4. **Synthesis**: This level should consider learning that is oriented towards the reconstruction of new knowledge structures or the products of the analysis of their different elements. Activities in this level are defined by the presence of a creative process that allows the integration of recently learned information in new structures or schemas.
5. **Assessment**: This level considers the activities that are aimed at assessing situations and decisions in a conscious and reasoned way.

**Description of the methodology**

**Phase 1. Competencies and topic mapping**

Mapping is carried out with the specific competencies of the course and the themes of the first level. One competency can cover more than one theme and does not necessarily have to be consecutive. Similarly, we found that one theme could contribute to various competencies.

|  | Theme 1 | Theme 2 | Thee 3 | Theme 4 |
| --- | --- | --- | --- | --- |
| Competency 1 | x | x |  |  |
| Competency 2 |  | x |  | x |

Additionally, during this exercise, two scenarios can be presented: a competency that is not in the syllabus or a theme that does not contribute to the development of any competency. The relevance of the missing element should be valued; it can be included in any of the existing elements (competencies/themes) or it can be removed.

|  | Theme 1 | Theme 2 | Theme 3 | Theme 4 |
| --- | --- | --- | --- | --- |
| Competency 1 |  | x |  |  |
| Competency 2 |  | x |  | x |
| Competency 3 |  | x | x |  |
| Competency 4 |  |  |  |  |

As a final result, we can be certain that there is a correspondence between learning outcomes and content.

**Phase 2. Location of the cognitive level of the competencies**

This second phase involves the assessment of the competencies with respect to the final cognitive level they affect. If the cognitive level of analysis is important, we must ensure that previous cognitive levels are considered within the activity or in new activities. This requires working in a double-entry matrix in which the left column corresponds to the competencies and the top row indicates the cognitive level. This provides a guideline of the expected cognitive level.

|  | Level 1 | Level 2 | Level 3 | Level 4 | Level 5 |
| --- | --- | --- | --- | --- | --- |
| Competency 1 |  |  | x |  |  |
| Competency 2 |  |  |  | x |  |
| Competency 3 |  | x |  |  |  |
| Competency 4 |  |  |  | x |  |

**Phase 3. Definition of learning experiences and their results according to the cognitive level**

At the end of the previous phase, the work focused on reviewing the transverse cuts of the cube to verify where the learning activities and relevant results were located. Each cut corresponds to the cognitive levels. For example, the second cut corresponds to Level 2 (application), where the competency is located. This means that a learning activity must be generated to promote the activation of this cognitive process and its results should be consistent.

| Level 2 | Theme 1 | Theme 2 | Theme 3 | Theme 4 | Theme 5 |
| --- | --- | --- | --- | --- | --- |
| Competency 1 |  |  |  |  |  |
| Competency 2 |  |  |  |  |  |
| Competency 3 |  | x |  |  |  |
| Competency 4 |  |  |  |  |  |

Each activity should be encoded with the following nomenclature:
Competency + theme + cognitive level. Example: C3T2CN2

It is important to note the following options: *1)* that the same competency in the same theme is addressed at two cognitive levels and *2)* that one competency is worked on in two or more themes and at more than one cognitive level.

After identifying the amount and depth of the required activities, it is important to ensure that the evidence is consistent with the expectations. If competency 2 is addressed in themes 3 and 4 at cognitive levels 2, 3 and 4 (application, analysis and synthesis), the evidence of learning should not be a written exam of recovery information but a project or scenario of a case that shows the level of the new learning.

**
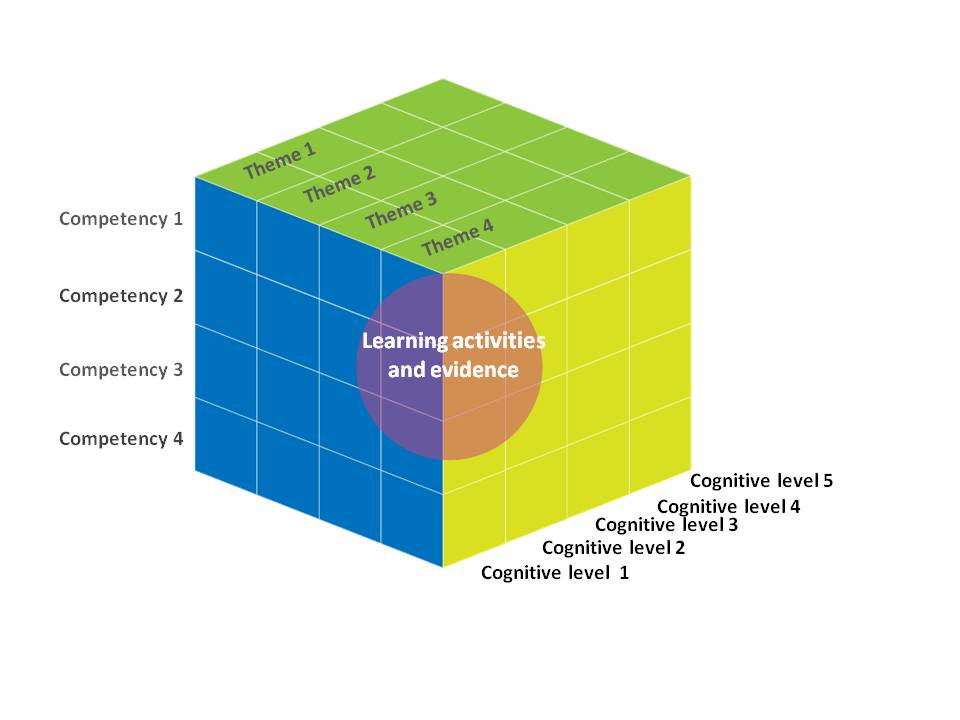
**
