# Supplementary material for: Training Primary Health Professionals in Breast Cancer Prevention: Evidence and Experience from Mexico
Source: J Cancer Educ. 2016 Jun 30;33(1):160–6. doi: 10.1007/s13187-016-1065-7 (PMC5762772; doi:10.1007/s13187-016-1065-7)
Supplement: Supplementary file 3 — (PDF 2580 kb) [file 13187_2016_1065_MOESM3_ESM.pdf]

Early Breast Cancer Detection

A Job for Everyone

Guide for trainers

List of competencies and of contents of Chapter 3

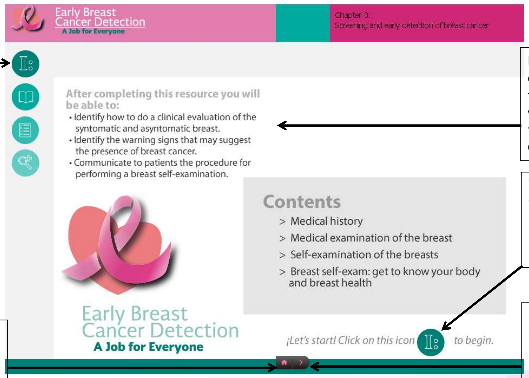

Introduction button

List of competencies that students will acquire at the end of Chapter 3

You must click this button to begin with the introduction of Chapter 3

Home
This button always brings you back to this screen

If you click on this button you can move forward through the following screens

Introduction to Chapter 3

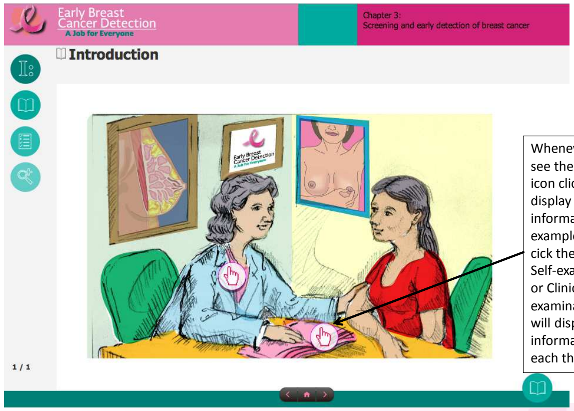

Whenever you see the hand icon click on to display more information. For example, if you cick the image of Self-examination or Clinical examination it will display more information of each theme.

Content of the specific interventions to detect breast cancer

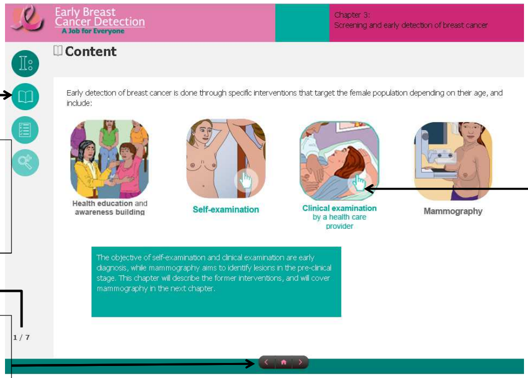

Content button

This number tells you in which screen of content you are now: one of seven

If you click on this button you can move backward through the last screens

Whenever you see the hand icon click on to display more information. For example, if you click the image of Self-examination or Clinical examination it will display more information of each theme.

Content of medical history

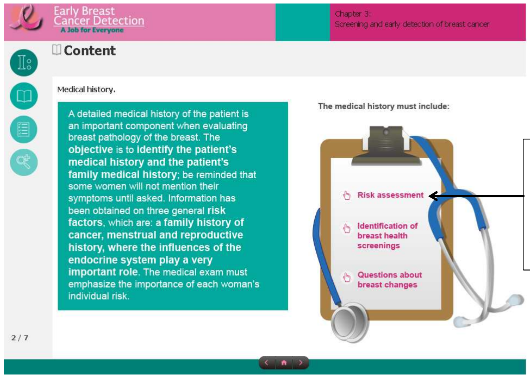

Click on each of the elements that the medical history must include to display more information

Content of medical examination of the breast

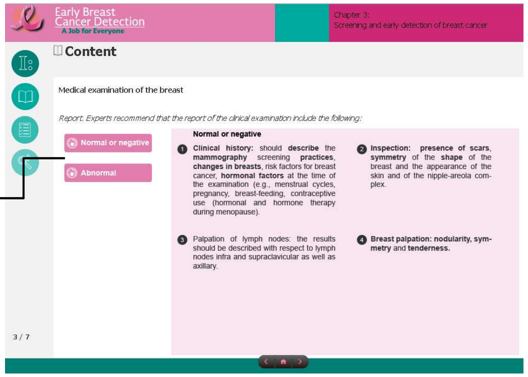

Click on each text box to display more information

Content of how to perform a medical examination of the breast

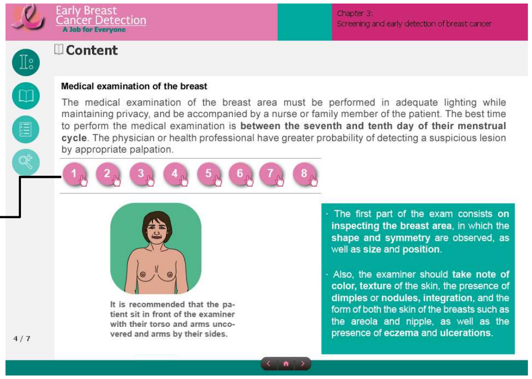

Click on each number to display more information

Content of how to interpret the medical examination of the breast

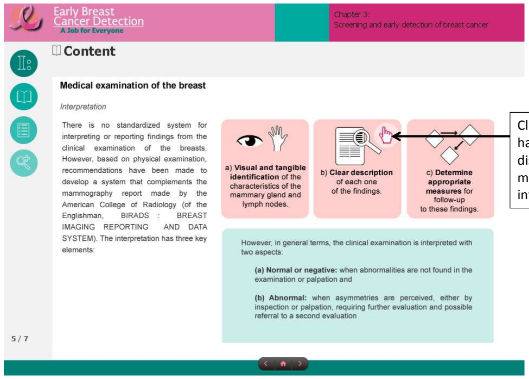

Click on the hand icon to display more information

Content of self-examination of the breasts

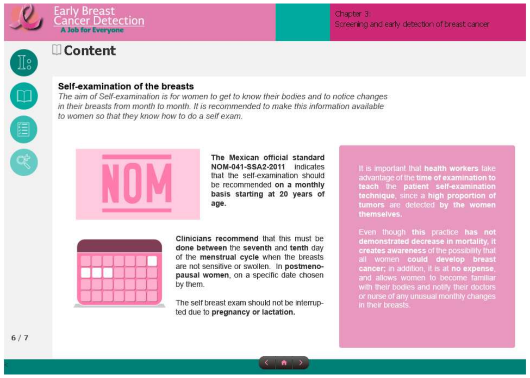

Content of the process to realize a self-examination of the breasts

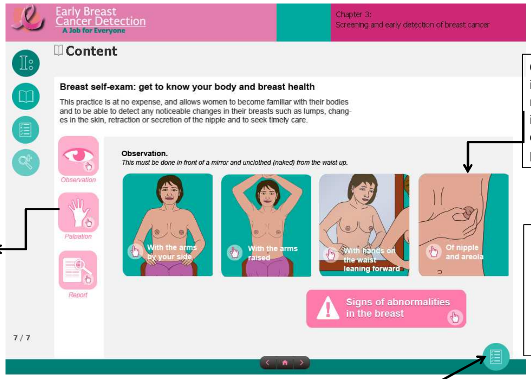

Click on each icon to display more images of the process to realize a self-examination of the breasts

Click on each image to display more information of each step of the process

Remember that every time you see the hand icon if you click on it more information will display

This icon indicates you that evaluation starts at the next screen

Evaluation of the content

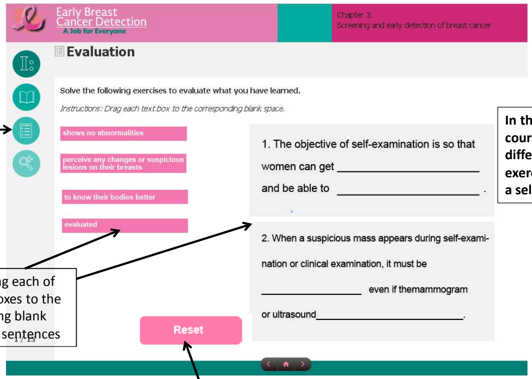

Evaluation button

In this section of the course, you will find different kinds of exercises to perform a self evaluation.

Click and drag each of these text boxes to the corresponding blank space of the sentences

To start again the evaluation click this button

Evaluation of the content of clinical breast exam

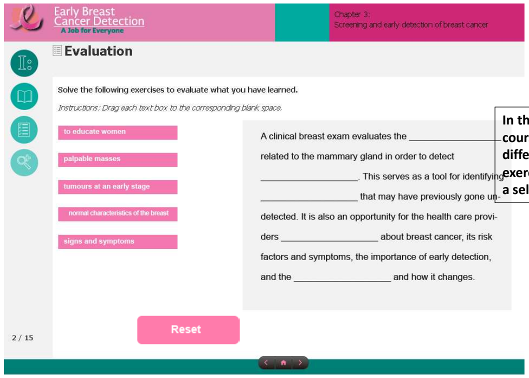

In this section of the course, you will find different kinds of exercises to perform a self evaluation.

Drag and drop evaluation

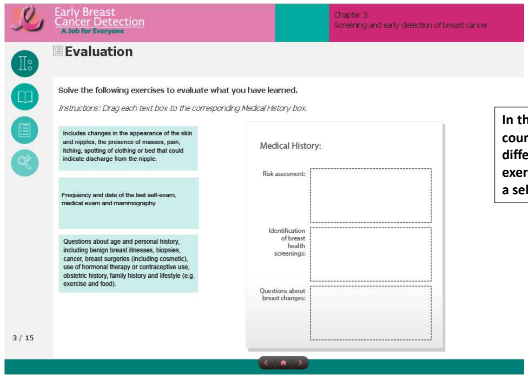

In this section of the course, you will find different kinds of exercises to perform a self evaluation.

Clinical breast examination

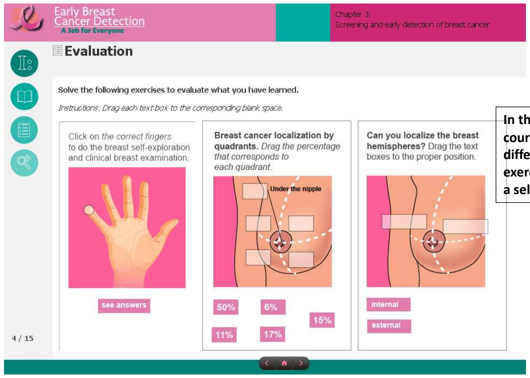

In this section of the course, you will find different kinds of exercises to perform a self evaluation.

Drag and drop evaluation

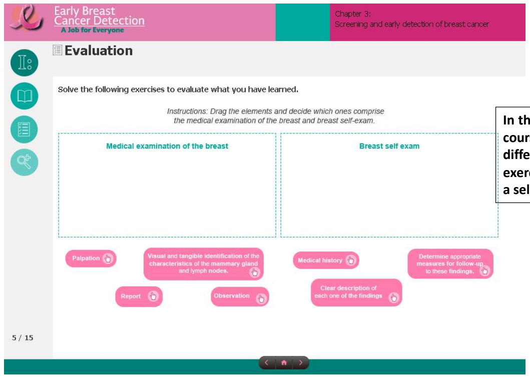

In this section of the course, you will find different kinds of exercises to perform a self evaluation.

Multiple choice evaluation

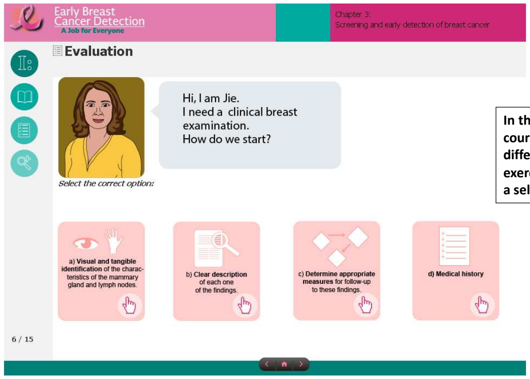

In this section of the course, you will find different kinds of exercises to perform a self evaluation.

Drag and drop evaluation

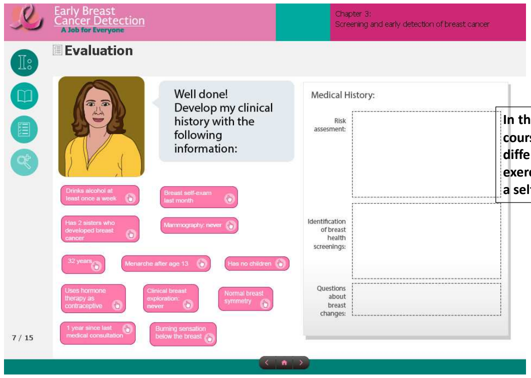

In this section of the course, you will find different kinds of exercises to perform a self evaluation.

Multiple choice evaluation

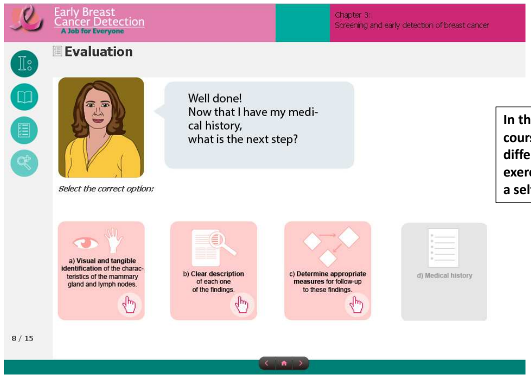

In this section of the course, you will find different kinds of exercises to perform a self evaluation.

Drag and drop evaluation

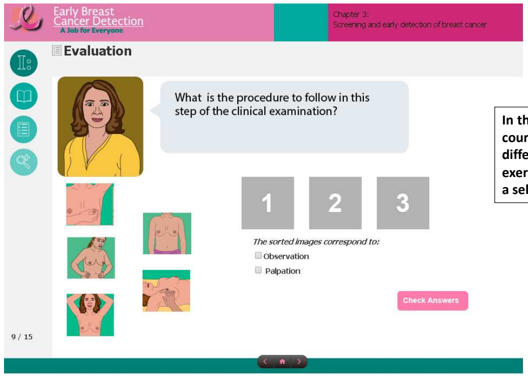

In this section of the course, you will find different kinds of exercises to perform a self evaluation.

Drag and drop evaluation

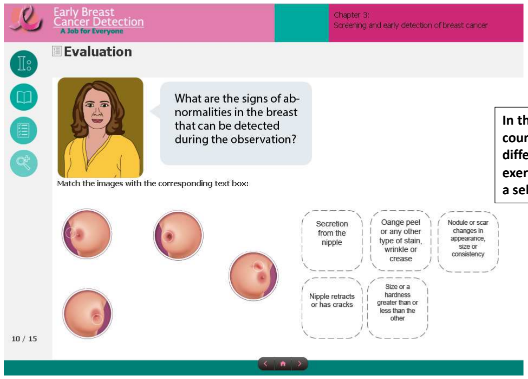

In this section of the course, you will find different kinds of exercises to perform a self evaluation.

Multiple choice evaluation

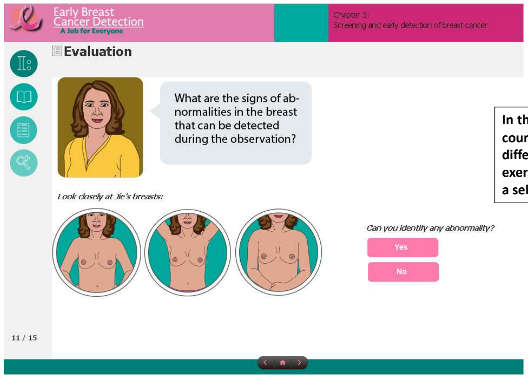

In this section of the course, you will find different kinds of exercises to perform a self evaluation.

Multiple choice and image interactive evaluation

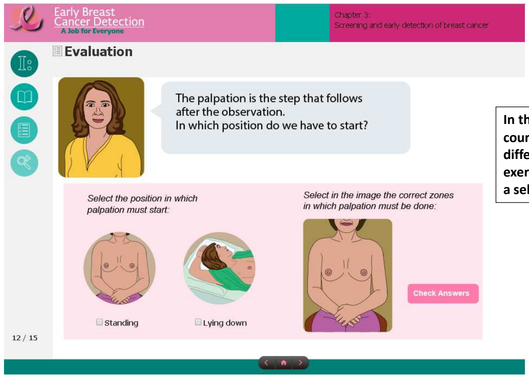

In this section of the course, you will find different kinds of exercises to perform a self evaluation.

Multiple choice evaluation

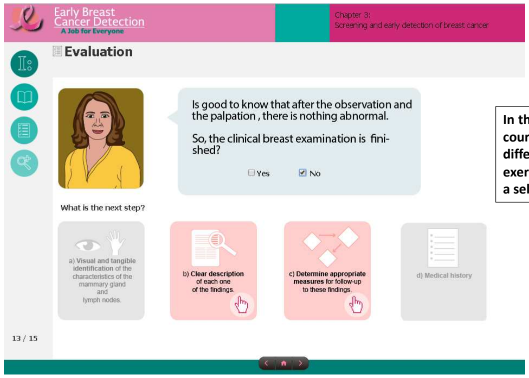

In this section of the course, you will find different kinds of exercises to perform a self evaluation.

Drag and drop evaluation

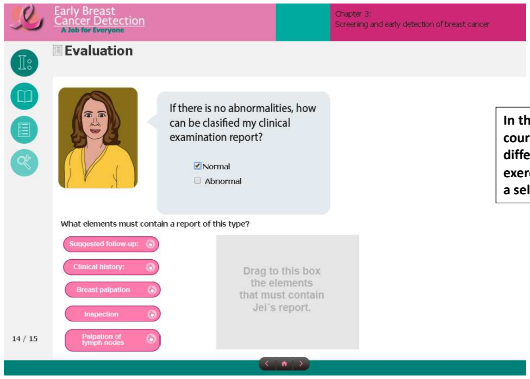

In this section of the course, you will find different kinds of exercises to perform a self evaluation.

Multiple choice evaluation

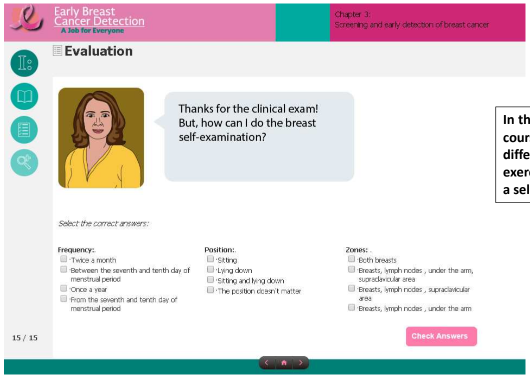

In this section of the course, you will find different kinds of exercises to perform a self evaluation.

Instituto Nacional de Salud Pública

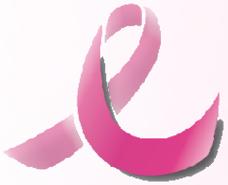

Early Breast Cancer Detection

A Job for Everyone
